# 抗体偶联药物在肺癌中的研究进展与展望

**DOI:** 10.3779/j.issn.1009-3419.2022.102.22

**Published:** 2022-07-20

**Authors:** 以香 朱, 志杰 王

**Affiliations:** 100021 北京，国家癌症中心，国家肿瘤临床医学研究中心，中国医学科学院北京协和医学院肿瘤医院内科 CAMS Key Laboratory of Translational Research on Lung Cancer, State Key Laboratory of Molecular Oncology, Department of Medical Oncology, National Cancer Center/National Clinical Research Center for Cancer/Cancer Hospital, Chinese Academy of Medical Sciences Peking Union Medical College, Beijing 100021, China

**Keywords:** 肺肿瘤, 抗体偶联药物, 精准治疗, Lung neoplasms, Antibody drug conjugates (ADCs), Precision medicine

## Abstract

抗体偶联药物（antibody drug conjugates, ADCs）是一类将特异性单克隆抗体与细胞毒性药物通过接头偶联形成的新型抗肿瘤药物，不仅在乳腺癌、血液系统肿瘤等多个癌种中取得了显著效果，在肺癌中也初见成效。本文通过对ADC的作用原理，在肺癌中的应用，以及面临的问题和挑战等方面进行概述，希望能够给临床工作一些参考。

## Abstract (en)

Antibody drug conjugates (ADCs) are a novel class of anti-cancer drugs, which combined the specificity of monoclonal antibodies with the cytotoxic palyload via the linkers. Many ADCs have not only verified impressive activity in a variety of cancers, including breast cancer and hematological system tumors, but also in lung cancer. The aim of this study was to provide informations for practice by summarizing the mechanism of action, clinical application and problems and challenges of ADCs.

国家癌症中心统计数据^[1]^显示，肺癌仍是我国总体发病率和癌症相关死亡率最高的恶性肿瘤。根据病理类型分为非小细胞肺癌（non-small cell lung cancer, NSCLC）和小细胞肺癌（small cell lung cancer, SCLC）。尽管靶向治疗和免疫治疗显著改善了患者的预后，仍有部分患者无法从中获益；即便获得最初的疗效，亦不可避免发生耐药，因此迫切需要寻找新型安全、有效的治疗方法。抗体偶联药物（antibody drug conjugates, ADCs）是一类由单克隆抗体与细胞毒性载荷通过链接子偶联组成的新型药物。最初的设计源于Pual教授的“魔法子弹”学说，即将毒性药物安装在特异性靶向肿瘤细胞的载体上，既可以实现精准杀伤肿瘤又不损害正常细胞^[2]^。早期的ADCs受限于药代动力学和药物学的特性以及载荷效力不足等因素，未能获得良好疗效^[3, 4]^。随着生物科技的发展，已成功开发出多种新型的ADCs药物，并在多种恶性肿瘤中取得显著成效。本文将从ADCs的作用原理，在肺癌的最新研究进展及面临的问题和挑战等方面进行综述。

## 1 ADCs的作用原理

ADCs主要由抗体、接头和细胞毒性载荷三部分组成，通过接头将细胞毒性载荷与靶向肿瘤细胞表面抗原的抗体相连接，然后将细胞毒性药物传递到肿瘤细胞内起到抗肿瘤效果。

### 1.1 抗体

抗体与表达目标抗原的靶细胞结合为ADCs提供了治疗窗口。理想的抗体应具有对靶抗原的高度特异性和亲和力、长半衰期和低免疫原性等特点。人源化或嵌合免疫球蛋白G（immunoglobulin G, IgG）因免疫原性低、亲和力高和半衰期长，成为ADCs最常用的抗体骨架^[5]^。此外，IgG还可以通过介导抗体依赖性细胞毒作用（antibody-dependent cellular cytotoxicity, ADCC）发挥次级免疫功能。相比于IgG2和IgG4，IgG1的半衰期更长、ADCC作用更强，成为ADC抗体的最佳选择^[6]^。此外，理想的抗体靶点应在肿瘤细胞高表达或特异性表达，正常细胞低表达甚至不表达，这样可以实现最大化地杀伤靶细胞，减少全身毒性反应。

### 1.2 接头

接头的作用是将抗体与细胞毒性载荷进行偶联^[7]^，应具有较好的稳定性，避免ADCs到达靶细胞前过早释放细胞毒性载荷；一旦被内化，能迅速有效地释放细胞毒性药物发挥抗肿瘤作用^[8]^。目前ADCs有2种类型的接头，即可切割型和不可切割型。前者指在肿瘤细胞内快速分解并释放细胞毒性载荷，但稳定性差；后者形成不可还原键，依赖抗体-连接结构的溶酶体降解，只有完全降解后才能释放细胞毒性载荷^[9]^。两者有各自的优缺点，后者虽然稳定性强，可以减少脱靶毒性的风险，但可能会导致载荷上保留带电的氨基酸，影响其功能和细胞渗透性^[10]^。

### 1.3 细胞毒性载荷

细胞毒性载荷是ADCs的“弹头”或“有效载荷”，要求在血液中最好是非免疫原性、低毒，并能在皮摩尔或纳摩尔浓度下发挥效果^[6]^。早期的ADCs应用常规化疗药物作为载荷，但因抗体渗透性低、载荷效力不足，无法发挥预期效果^[11]^。目前应用的主要是高效细胞毒性的药物，如微管抑制剂、DNA切割剂和拓扑异构酶抑制剂等^[12]^。除有效载荷外，药物与抗体的比率和有效载荷的疏水性也会影响细胞毒性药物发挥作用，前者可以影响药物分布及药代动力学，后者可以影响ADCs的“旁观者效应”^[13]^。ADCs释放细胞毒性药物可以从表达抗原的细胞扩散到邻近抗原阴性的细胞，诱导其发生细胞毒作用。这种对抗原阴性细胞的额外功效称为“旁观者效应”。

综上，ADCs的经典作用机制来源于单克隆抗体与靶抗原结合，引起ADC内吞进入细胞，之后接头分解引起细胞毒性载荷释放，发挥对靶细胞的杀伤作用^[14]^。此外，旁观者效应也是潜在的作用机制。

## 2 ADCs在肺癌中的研究进展

目前针对肺癌的ADCs主要有：靶向人表皮生长因子受体2（human epidermal growth factor receptor 2, HER2）、人表皮生长因子受体3（human epidermal growth factor receptor 3, HER3）、人滋养细胞表面抗原2（trophoblast cell-surface antigen 2, TROP2）、癌胚抗原相关细胞附着分子5（carcinoembryonic antigen-related cell adhesion molecule 5, CEACAM5）、细胞间质上皮转换因子（cellular-mesenchymal epithelial transition factor, c-MET）和Delta样蛋白3（delta-like protein 3, DLL3）等靶点的ADCs。

### 2.1 NSCLC

#### 2.1.1 HER2

HER2是由*ERBB*2基因编码的一种酪氨酸激酶受体，属于表皮生长因子受体（epidermal growth factor receptor, EGFR）家族成员之一，与其他家族成员形成同源或异源二聚体后引起受体酪氨酸残基磷酸化，启动MAPK、PI3K/AKT等多种信号通路激活，促进癌症发生^[15]^。HER2在NSCLC中的变异形式包括突变（2%-4%）、扩增（1%-4%）和过表达（20%）^[12, 16]^。Trastuzumab Emtansine（T-DM1）是由抗HER2的单抗曲妥珠单抗与微管抑制剂Emtansine（DM1）通过不可切割的接头偶联形成的新型ADC，药物抗体偶联比（antibody-drug conjugate, DAR）为3.5^[17]^。Ⅱ期研究纳入了49例*HER2*突变或扩增型晚期NSCLC，T-DM1治疗的总有效率（overall response rate, ORR）为51%，中位无疾病进展生存期（progression-free survival, PFS）为5个月，其中*HER2*突变、扩增和同时存在者的ORR分别为55%、50%和50%。治疗相关不良反应（adverse event, AE）多为1级-2级，包括：谷草转氨酶（aspartate transaminase, AST）/谷丙转氨酶（alaninetransaminase, ALT）升高（63%）、血小板减少（31%）、恶心（29%）、乏力（16%）和输液反应（14%）等，仅2例患者出现了3级AE，分别为贫血和血小板减少^[18]^。T-DM1在HER2过表达的NSCLC中作用有限，Ⅱ期研究因疗效不佳提前终止，仅1例*HER2*突变患者观察到部分缓解（ORR: 6.7%, 1/15），8例经免疫组织化学染色（immunohistochemistry staining, IHC）证实为HER2过表达或经荧光原位杂交（fluorescence *in situ* hybridization, FISH）证实*HER2*扩增者均无应答，报道的3级-4级AE发生率较前者高，主要为血小板减少（40%）和肝损伤（20%），还有1例急性肾衰竭（7%）^[19]^。另一项探讨HER2过表达的Ⅰ期研究^[20]^纳入了49例经治的晚期NSCLC，HER2 IHC2+和3+的ORR分别为0和20%，中位PFS分别为2.6个月和2.7个月，无新发AE。

Trastuzumab Deruxtecan（T-DXd）是由人源化抗HER2单抗Trastuzumab与拓扑异构酶I抑制剂Deruxtecan（DXd）经可切割的四肽接头偶联形成的新型ADC，DAR为8^[21]^。Ⅱ期研究（DESTINY-Lung01）纳入了91例既往治疗失败的*HER2*阳性晚期NSCLC，其中*HER2*突变42例，HER2过表达49例，所有患者均接受T-DXd单药治疗，突变组的ORR和PFS分别为61.9%和14.0个月，过表达组分别为24.5%和5.4个月，两组≥3级AE发生率相似（52.4%和55.1%）^[22, 23]^。前者≥3级AE包括：中性粒细胞减少（26.2%）和贫血（16.7%），此外，5例（11.9%）患者发生了2级药物相关的间质性肺病（interstitial lung disease, ILD）；25例（59.5%）患者需要中断治疗、16例（38.1%）需要减量以及10例（23.8%）治疗终止；后者≥3级AE有：中性粒细胞减少（20.4%）和乏力（10.2%），还有8例（16.3%）患者发生了药物相关的ILD，其中3例为致死性ILD（6.1%）；有26例（53.1%）、17例（34.7%）和11例（22.4%）患者分别需要中断治疗、降低剂量和停止治疗。后续更新的针对91例*HER2*突变晚期NSCLC的分析^[24]^结果显示，ORR为55%，中位PFS和OS分别为8.2个月和17.8个月。鉴于上述研究，T-DM1和T-DXd被美国国立综合癌症网络（National Comprehensive Cancer Network, NCCN）推荐用于治疗经标准治疗失败的*HER2*突变型晚期NSCLC。综上可见，虽然T-DM1和T-DXd在*HER2*突变型晚期NSCLC中具有良好疗效，但在过表达的患者中作用有限，需进一步区分不同的分子类型，探索获益人群。并且，上述结果均来自Ⅰ期-Ⅱ期的单臂研究，仍需更多研究证实。

#### 2.1.2 HER3

HER3是EGFR家族的另一成员，虽然缺乏激酶活性，但可以与其他家族成员形成同源或异源二聚体引起受体酪氨酸残基磷酸化，激活PI3K/AKT、MAPK等多个信号通路，引起癌症发生^[25]^。研究显示，19%的NSCLC存在HER3过表达，腺癌可达46%^[25]^，而且HER3还介导了EGFR-酪氨酸激酶抑制剂（tyrosine kinase inhibitor, TKI）的耐药^[26]^。Patritumab Deruxtecan（U3-1402）是由人源化抗HER3的单抗Patritumab与拓扑异构酶I抑制剂DXd经可切割的四肽接头偶联形成的新型ADC，DAR约为8^[27]^。Ⅰ期研究^[28]^入组了56例奥希替尼耐药或第一/二代EGFR-TKI进展后无T790M突变的晚期NSCLC，几乎所有患者均表达HER3。结果显示，U3-1402单药的ORR为25%，中位持续缓解时间（duration of response, DOR）为7个月，≥3级AE主要为血小板减少（25%）和中性粒细胞减少（16%）。Ⅱ期研究正在进行中。虽然相比于其他靶向药，U3-1402的疗效并不显著，但是对三代TKI耐药及不适合三代TKI的患者来说，有可能是一种新的治疗方案。

#### 2.1.3 TROP2

TROP2是一种细胞表面糖蛋白，与细胞迁移和增殖有关，在多种上皮源性肿瘤中高表达，其中肺腺癌、肺鳞癌和肺高级别神经内分泌癌的比例分别为64%、75%和18%^[29]^。Datopotamab Deruxtecan（DS-1062）是由人源化抗TROP2的单抗与拓扑异构酶I抑制剂DXd经可切割的四肽接头偶联形成的新型ADC，DAR为4^[30]^。在Ⅰ期研究中入组了175例复发/难治的晚期NSCLC患者，结果显示，DS-1062 4 mg/kg、6 mg/kg和8 mg/kg的ORR分别为23%、21%和25%，中位PFS分别为4.3个月、8.2个月和5.4个月，治疗相关AE与剂量成依赖性关系，≥3级AE发生率为10%-34%，包括：口腔炎、恶心、疲劳、黏膜炎和贫血等；其中，8 mg/kg组发生了4例≥3级的ILD^[30]^。因此，后续研究的推荐剂量为6 mg/kg。目前，针对驱动基因阳性的晚期NSCLC的Ⅱ期研究（NCT04484142）和既往治疗失败的NSCLC的Ⅲ期临床研究（NCT04656652）正在开展，希望能为复发/难治NSCLC带来获益。

Sacituzumab Govitecan（IMMU-132）是由人源化抗TROP2单抗与拓扑异构酶I抑制剂Govitecan（SN-38）通过可切割接头偶联形成的新型ADC^[31]^。一项多中心单臂研究^[31]^纳入了54例既往治疗失败的晚期NSCLC，其中91%的患者至少接受过2种治疗，IMMU-132在总人群的ORR为19%，中位DOR为6.0个月，意向治疗分析人群的ORR为17%，中位PFS和OS分别为5.2个月和9.5个月，≥3级AE发生率较低，主要为中性粒细胞减少（28%）、腹泻（7%）、恶心（7%）和粒细胞减少伴发热（4%）。在针对生物标志物的分析中，TROP2 IHC阳性（2+/3+）未能观察到更好的疗效。虽然IMMU-132的整体有效率不高，但对于接受过多重治疗的患者而言，该药安全性良好，且PFS和OS令人鼓舞。

#### 2.1.4 CEACAM5

CEACAM5即CD66e，是一种癌胚抗原基因编码的糖蛋白，在乳腺癌、NSCLC等多个癌种呈中-高水平表达，在正常组织呈低表达，其中20%的非鳞NSCLC呈高表达（> 50%），25%呈中等表达（1%-49%）^[32, 33]^。SAR408701是由人源化抗CEACAM5单抗与微管抑制剂美登素DM4通过可切割四肽接头偶联形成的新型ADC，DAR为3.9^[32]^。在首项临床研究中入组了92例既往治疗失败的晚期NSCLC患者，SAR408701在CEACAM5中等表达组的ORR仅为7.1%，高表达组的ORR达20.3%，中位DOR为5.6个月^[33]^。≥3级AE的发生率为47.8%，与药物相关仅为15.2%，为角膜炎（10.9%）和乏力（4.3%）；最严重的AE为与疾病进展有关的呼吸困难。针对一线化疗和免疫治疗进展伴EACAM5高表达的晚期NSCLC的Ⅲ期临床研究（NCT02187848）正在开展，期待能有好的临床获益。

#### 2.1.5 c-MET

c-MET是由*MET*基因编码的一种受体酪氨酸蛋白激酶，又称肝细胞生长因子受体，与其配体结合后可以激活PI3K/AKT、MAPK等多个信号通路，与细胞增殖、迁移和侵袭密切相关^[34]^。在NSCLC中的变异形式包括：突变、扩增和过表达，其中*MET 14*外显子跳跃突变的发生率为3%-4%；*MET*原发扩增的发生率约为3%，继发扩增为10%-15%且与多种TKI耐药有关；MET蛋白过表达的发生率约24%^[12, 34, 35]^。Telisotuzumab Vedotin（ABBV-399）是由人源化抗c-MET单抗与微管抑制剂Auristatin E通过可切割接头偶联形成的新型ADC，DAR约为3.1^[36]^。Ⅰ期研究^[37]^显示，ABBV-399单药或联合厄洛替尼治疗c-MET表达阳性的NSCLC的ORR为18.8%。针对c-MET阳性鳞状NSCLC的Ⅱ期研究SWOG S1400K因疗效不佳提前关闭，可分析的数据显示ABBV-399在未经免疫治疗的患者中疗效似乎更好一些，ORR、PFS和OS分别为9%、2.4个月和5.6个月；接受过免疫治疗者分别为0%、1.6个月和4.8个月^[38]^。ABBV-399在非鳞NSCLC中的疗效令人鼓舞。Ⅱ期研究^[39]^显示，ABBV-399治疗c-MET阳性伴EGFR野生型的非鳞NSCLC的ORR为35.1%，其中c-MET高表达组为53.8%，中等表达组为25%；但在*EGFR*突变和鳞状NSCLC组中获益有限，ORR分别为13.3%和14.3%。≥3级AE的发生率为44%，主要为肺炎（5%）和肿瘤进展（4%）。综上可见，ABBV-399在复发/难治性c-MET高表达伴*EGFR*突变阴性的非鳞NSCLC中疗效较为突出，但在EGFR突变和鳞状NSCLC中作用有限，期待更多的研究加以证实。SHR-A1403是首个进入临床阶段的国产c-MET ADC，由人源化抗c-MET单抗与微管抑制剂通过不可切割接头偶联而成，DAR为2.1^[40]^。临床前研究显示SHR-A1403可以克服c-MET高表达细胞对奥希替尼的耐药，但目前缺乏SHR-A1403治疗NSCLC的临床数据。

#### 2.1.6 蛋白酪氨酸激酶7（protein tyrosine kinase 7, PTK7）

PTK7是一种缺乏酪氨酸激酶活性的酪氨酸激酶受体蛋白，在NSCLC等人源性移植瘤模型的肿瘤初始细胞中富集，有可能成为潜在治疗靶点^[41]^。Cofetuzumab Pelidotin（PF-06647020）是由人源化抗PTK7的单抗与微管抑制剂Aaur0101通过可切割的接头偶联形成的新型ADC，DAR为4^[42]^。Ⅰ期研究纳入了31例经标准治疗失败的NSCLC，接受三周方案治疗者的ORR为16%，中位PFS和DOR分别为2.9个月和5.7个月^[42]^。此外，基线组织中PTK7高表达的患者较低表达者肿瘤退缩更明显（*P*=0.027）。安全性分析显示PF-06647020的耐受性好，在所有接受三周方案的泛瘤种患者中，≥3级AE的发生率为39.3%，包括：中性粒细胞减少（25%）、头痛（4.5%）、恶心（3.6%）和乏力（2.7%）等。因此，后续或可以开展针对PTK7作为生物标志物的探索。

### 2.2 SCLC

#### 2.2.1 DLL3

DLL3是NOTCH补体家族成员之一，在72%的初治以及85%复发的SCLC肿瘤组织呈高表达，正常组织呈低表达，可以成为SCLC治疗的潜在靶点^[43]^。Rovalpituzumab Teserine（Rova-T）是由人源化抗DLL3的单抗与抗肿瘤抗生素SC-DR002通过可切割接头偶联形成的新型ADC，DAR为2^[44]^。Ⅰ期研究纳入了74例复发/难治性SCLC，在60例可评估疗效的患者中，Rova-T的ORR为18%，DCR为68%，中位DOR和PFS分别为5.6个月和2.8个月^[44]^。值得注意的是，DLL3高表达者（≥50%）的ORR和DCR可达38%和88%，中位DOR和PFS分别为4.4和4.3个月。≥3级AE发生率为38%，主要是血小板减少（12%）、浆膜腔积液（11%）和皮肤反应（8%）。然而Ⅱ期和Ⅲ期研究均以失败告终，Ⅱ期研究（TRINITY）纳入了339例至少接受过2线治疗的SCLC，Rova-T治疗的ORR和中位OS仅为12.4%和5.6个月，即使DLL3高表达人群也未观察到更好的疗效，≥3级AE发生率为63%（40%与药物相关），5级AE发生率为10%（3%与药物相关），其中与药物相关的≥3级AE有：血小板减少（11%）、光过敏（7%）、贫血（4%）和胸腔积液（4%）等^[45]^。Ⅲ期研究（TAHOE）比较了Rova-T和托泊替康作为二线治疗的效果，因Rova-T的疗效较对照组差而终止，两者的ORR分别为15% *vs* 21%，中位PFS为3.0个月 *vs* 4.3个月（HR=1.51, 95%CI: 1.22-1.87），中位OS为6.3个月 *vs* 8.6个月（HR=1.46, 95%CI: 1.17-1.82），≥3级AE分别为64% *vs* 88%，与治疗相关者分别为56% *vs* 57%^[46]^。即使作为标准方案治疗后的维持治疗和联合化疗作为一线治疗，Rova-T均未能观察到获益^[47-48]^。此外，一项Ⅰ期-Ⅱ期研究^[49]^还对Rova-T与免疫检查点抑制剂的联合模式进行了探索。共入组了42例既往治疗失败的广泛期SCLC，采用Rova-T+纳武利尤单抗±小剂量伊匹木单抗治疗。结果显示，尽管提高了既往治疗失败的广泛期SCLC的有效率（ORR=30%），但整体安全性差，患者均出现了至少1次治疗相关性AE，还有3例5级AE，分别为肺炎（*n*=2）和急性肾衰竭（*n*=1）。DLL3高表达（> 75%）与非高表达（25%-75%）患者的有效率、PFS和OS相似。SC-002也是由人源化抗DLL3的单抗与SC-DR002通过可切割接头偶联形成的ADC，DAR为2^[50]^。Ⅰ期研究^[50]^纳入了35例复发/难治性SCLC或大细胞神经内分泌肿瘤，ORR仅为14%（5/35），即使在DLL3表达阳性者ORR也只有11.8%。综上可见，针对DLL3的ADCs研究均以失败告终。

#### 2.2.2 CD56

CD56，又称神经细胞粘附分子，是神经内分泌肿瘤的特异性标志物，在SCLC的阳性率为100%^[51]^。Lorvotuzumab Mertansine（LM, IMGN901）是由人源化抗CD56单抗与DM1通过可切割的接头偶联形成的新型ADC^[52]^。Ⅰ期/Ⅱ期研究^[53]^分别纳入了33例和50例复发/难治性广泛期SCLC，LM联合化疗对比单纯化疗并不能改善患者的预后，中位PFS分别为6.2个月 *vs* 6.7个月（HR=0.93, 95%CI: 0.58-1.51），中位OS分别为10.1个月 *vs* 11.0个月（HR=0.83, 95%CI: 0.48-1.52）。3级-4级AE发生率较高，Ⅰ期和Ⅱ期分别为88%和70%，还有21例患者出现了治疗相关死亡。Ⅰ期和Ⅱ期研究中≥3级治疗相关AE的发生率相似，主要包括：贫血、周围神经毒性、中性粒细胞减少和血小板减少等。

#### 2.2.3 TROP2

Sacituzumab Govitecan（SG）是由人源化抗TROP2的单抗与SN-38通过独特可水解接头偶联而成的新型ADC，DAR为7.6^[54]^。一项评估了SG在既往治疗失败的转移性SCLC的单中心研究^[55]^显示出良好的耐受性和疗效，ORR为14%，中位PFS和OS分别为3.7个月和7.5个月，≥3级AE主要为中性粒细胞减少（34%）、乏力（13%）、腹泻（9%）和贫血（6%）等。在可获得组织样本的25例患者中，TROP2高表达（IHC 2-3+）与低表达（IHC 0-1+）组的PFS和OS无差异。Ⅰ期/Ⅱ期研究^[56]^纳入了62例复发/难治性SCLC，ORR为17.7%，中位DOR和OS分别为5.7个月和7.1个月，≥3级AE主要是中性粒细胞减少。尽管有效率不高，但ADC药物在复发/难治性SCLC中安全性良好，且具有一定抗肿瘤活性，或可以开展联合模式及更多分子标志物探索。

### 2.3 ADCs的联合探索

在提高ADCs疗效的探索上，联合治疗已初见成效，比如：与抗血管生成药物的联合有可能通过改善肿瘤血管环境来改善ADCs向肿瘤组织的传递；与小分子激酶抑制剂联合可以刺激抗原内化，激活ADC内吞作用和活性；与免疫检查点抑制剂联合，可以上调树突状细胞表达、增加CD8^+^T细胞浸润等提高免疫治疗的疗效；与其他抗肿瘤药物联合通过互补或协同作用增加有效载荷的活性等^[13]^。一项入组了66例经铂类治疗失败的卵巢癌的Ⅰ期研究^[57]^显示，Mirvetuximab Soravtansine（一种人源性抗叶酸受体α单抗与微管抑制剂DM4经可切割接头偶联形成的ADC）联合贝伐珠单抗具有较好的抗肿瘤活性，ORR为39%，中位PFS为6.9个月，特别是在既往未接受过贝伐珠单抗治疗，且既往治疗线数 < 3，伴叶酸受体α阳性的患者，ORR可达56%，PFS达9.9个月；而且≥3级AE发生率低，主要是：高血压（13.6%）、AST升高（6.1%）、ALT升高（4.5%）和血小板减少（4.5%）。BEGONIA研究对ADC联合免疫检查点抑制剂的疗效与安全性进行了探索，初步分析显示DS-1062联合度伐利尤单抗一线治疗未经选择的晚期三阴性乳腺癌的ORR高达74%，≥3级AE仅有4例口腔炎（14%）^[58]^。遗憾的是，关于SCLC的探索结果并不理想。Rova-T+纳武利尤单抗±伊匹木单抗联合治疗虽然提高了既往治疗失败的广泛期SCLC的有效率，但安全性差，最后以失败告终^[49]^，后续研究需要针对如何优化联合模式的安全性进行探索。此外，临床前研究发现IMMU-132联合PARP抑制剂治疗三阴性乳腺癌具有协同效果，无论BRCA1/2状态如何，联合方案较单药方案均能提高移植瘤模型的肿瘤退缩率，并延长疾病进展时间^[59]^。目前进行的有关肺癌的ADC联合治疗的研究如表 1所示，期待后期结果公布。

**表 1:** 正在进行的有关肺癌的ADCs联合治疗研究 Currently ongoing trials for the combination regimen of ADCs with other drugs in lung cancer

Antigen target	ADC drug name	NCT number	Study agents	Study phase (*n*)	Study population	Primary endpiont
HER2	T-DXd	NCT04042701	T-DXd+Pembrolizumab	Ⅰ (115)	HER2-expressing locally advanced/metastatic breast cancer, and HER2-expressing or mutant locally advanced/metastatic NSCLC	DLT; MTD; ORR
CEACAM5	SAR408701	NCT04524689	SAR408701+Pembrolizumab+ Cisplatin/Carboplatin±Pemetrexed	Ⅱ(96)	CEACAM5+, advanced or metastatic, non squamous NSCLC with no *EGFR* sensitizing mutation or *BRAF* mutation or *ALK/ROS* alterations	DLT
AXL	BA3011	NCT04681131	BA3011 or BA3011+PD-1 inhibitor	Ⅱ(240)	Metastatic NSCLC with prior disease progression on a PD-1/PD-L1 inhibitor	ORR; Safety
TROP2	DS-1062	NCT04612751	DS-1062+Durvalumab±Carboplatin	ⅠB (68)	Advanced or metastatic NSCLC	DLT; MTD
HER2	T-DXd	NCT04686305	T-DXd+Durvalumab+Cisplatin/Carboplatin±Pemetrexed	ⅠB (136)	HER2 positive advanced and metastatic NSCLC	Safety
CEACAM5	SAR408701	NCT04394624	SAR408701+Ramucirumab	Ⅱ(36)	Pre-treated non squamous NSCLC with CEACAM5 expression	DLT; ORR
HER2	T-DM1	NCT03784599	T-DM1+Osimertinib	Ⅱ(58)	NSCLC with resistance to a first, second or third generation EGFR-TKI-treatment, and HER2-overexpression	Safety; ORR
HER3	U3-1402	NCT04676477	U3-1402+Osimertinib	Ⅰ (252)	Locally advanced or metastatic *EGFR*-mutant NSCLC with treatment with osimertinib at least six weeks	DLT; ORR; Safety
TROP2	SKB264	NCT05351788	SKB264+KL-A167± Cisplatin/Carboplatin	Ⅱ(110)	Locally advanced/metastatic NSCLC	Safety; ORR
ADC: antibody-drug conjugates; NCT: national clinical trial; N: number; HER2: human epidermal growth factor receptor 2; NSCLC: non-small cell lung cancer; CEACAM5: carcinoembryonic antigen-related cell adhesion molecule 5; DLT: dose-limiting toxicity; MTD: maximum tolerated dose; ORR: overall response rate; PD-1: programmed death 1; PD-L1: programmed death-ligand 1; TROP2: trophoblast cell-surface antigen 2; EGFR: epidermal growth factor receptor; TKI: tyrosine kinase inhibitor; HER3: human epidermal growth factor receptor 3; ALK: anaplastic lymphoma kinase.						

## 3 挑战与展望

虽然ADCs在肺癌中已初见成效，但依然存在很多问题和挑战。

### 3.1 提高ADCs效率

抗体帮助ADCs内化进入肿瘤细胞，是其发挥作用的关键步骤之一。因此，寻找与靶抗原具有高度特异性和亲和力的抗体十分重要。双靶点抗体不仅可以增加抗体的内化，还可以提高对肿瘤细胞的特异性，与单靶点抗体相比，双靶点抗体具有更好的抗肿瘤活性，有可能成为新的研究方向^[60, 61]^。另外，将传统的抗体骨架转化为“小型”多肽片段或单链可变片段，可以通过降低偶联物的分子量提高组织的渗透性和有效载荷的传输^[62, 63]^。此外，创新有效载荷可有助于提高抗肿瘤效果，比如：选择靶向或免疫治疗药物作为有效载荷^[13]^等。还有提高DAR亦是提高ADCs抗肿瘤效率的重要方法之一，临床前研究显示，Dolaflexin技术可以提高DAR进而诱导移植瘤的肿瘤退缩^[64]^。

### 3.2 克服耐药

ADC耐药机制十分复杂，可能的机制包括：①靶抗原下调、抗原表达缺失或抗原基因突变导致ADC与肿瘤细胞结合减少或无法结合；②内化途径缺失导致ADC无法内吞及转运；③溶酶体蛋白水解或酸化功能降低或溶酶体转运蛋白功能缺失，抑制接头裂解和有效载荷在肿瘤细胞内的释放；④胞浆因子如PLK1激酶活化阻止有丝分裂，或细胞周期蛋白RB1缺失导致细胞周期停滞；⑤肿瘤细胞中ABC转运蛋白上调导致有效载荷被肿瘤细胞直接转运排出细胞；⑥促凋亡蛋白Bak和Bax活性缺失或抗凋亡蛋白BCl-2和Bcl-x过度表达导致凋亡通路失调^[13, 65]^。然而，目前尚缺乏针对ADC耐药的有效治疗手段。通过联合其他药物、换用不同靶点的ADC或制定新的载荷药物等是潜在逆转耐药的手段。

### 3.3 寻找获益人群

由于肿瘤细胞表达的靶抗原具有高度异质性，并且可能是动态变化。因此，需要针对肿瘤组织表达的目标抗原选择合适的ADCs。现有的ADCs中，少部分药物在目标靶点富集性患者中显示出有前景的疗效，如T-DM1和T-DXd治疗*HER2*突变型晚期NSCLC的ORR可达55%。但对其他变异类型，如HER2过表达的NSCLC则作用有限^[18, 24]^。目前绝大多数在研的ADC均缺乏有效的预测疗效的生物标志物，未来需要开展相关研究探索甄选真正的获益人群。

### 3.4 毒副反应管理

尽管ADCs已展现出良好的前景，但很多研究都观察到了严重的AE，例如：DESTINY-Lung01研究中有34%的患者需减量，25%的患者因严重AE停药，还有5级ILD发生^[22, 23]^。研究^[13]^显示，ADCs相关AE主要与其作用机制相关，如正常细胞表达了靶细胞抗原导致非目标性杀伤；接头提前裂解发生脱靶毒性等。因此，仍需不断改进ADCs药物使其实现低毒、高效的目的。另外，需要充分认识ADCs的不良反应及处理方法，建立相应的安全性管理策略和评价方法。

## 4 总结

虽然ADCs药物在晚期肺癌，特别是既往治疗失败的肺癌中展示出了良好的效果和安全性，但距离最初的设计目标依然还有很长的路要走。如何改良ADCs使其实现高效、低毒、精准尚待更多的研究探索。随着生物技术的不断进步，相信不久的将来定能真正实现“魔法子弹”的设想。
